# Giardiavirus infection alleviates growth restriction and intestinal damage caused by the intestinal parasite *Giardia duodenalis*

**DOI:** 10.1186/s13071-025-06692-4

**Published:** 2025-02-24

**Authors:** Lu Li, Lili Cao, Qiankun Yang, Zhiteng Zhao, Jianqi Yuan, Shaoxiong Liu, Qinqin Jin, Jianhua Li, Xin Li, Xiaocen Wang, Nan Zhang, Weina Jiang, Pengtao Gong

**Affiliations:** 1 State Key Laboratory for Diagnosis and Treatment of Severe Zoonotic Infectious Diseases, Key Laboratory for Zoonosis Research of the Ministry of Education, Institute of Zoonosis, and College of Veterinary Medicine, Jilin University, Changchun, 130062 China; 2 Jilin Academy of Animal Husbandry and Veterinary Medicine, Changchun, 130062 China; 3 Deparment of Pathology, Qingdao Municipal Hospital, Qingdao, 266071 Shandong China

**Keywords:** Giardia duodenalis, Giardiavirus, Extracellular vesicles, EVs-coated GLV, Growth restriction, Intestinal damage

## Abstract

**Background:**

*Giardia duodenalis* is a prevalent intestinal pathogen causing giardiasis, a condition characterized by diarrhea and frequently linked to malnutrition and growth impairments in children. The virulence of Giardiavirus (GLV) may efficiently clear *Giardia* parasites from infected patients. However, we have a limited understanding of GLV transmission among *Giardia* species and GLV-infected *Giardia*’s impact on pathogenicity.

**Methods:**

This study investigated extracellular vesicles (EVs) isolated via ultracentrifugation or exosome assay kit to detect the presence of GLV in EVs, the results were detected using ultrastructure and molecular methods, including transmission electron microscopy, scanning electron microscopy, quantitative polymerase chain reaction (qPCR), and dot blot. Transwell migration assays confirmed the spread of GLV-enveloped EVs among *Giardia* species using inhibitor experiments and immunofluorescence. Mice gavaged with *Giardia*, with or without GLV infection, were assessed for disease progression, including growth parameters (weight and size gains), intestinal permeability, and pathology.

**Results:**

Parts of GLV exploit the *Giardia* EVs pathway to reach the extracellular environment, allowing GLV to spread among *Giardia* species via these EVs. The uptake of GLV-containing EVs by *Giardia* results in rapid trophozoite infection, and GLV wrapped in EVs also offers protection against external interference. Importantly, EV-coated GLV-infected *Giardia* leads to divergent clinical symptoms in mice, posing less risk to mice and reducing symptoms, such as emaciation, stunted growth, and lesion damage, compared with GLV-free *Giardia*-infected mice.

**Conclusions:**

Our studies show that GLV wrapped in EVs can spread among *Giardia* species, and GLV infection alleviates the lesions caused by *Giardia*. These findings reveal that GLV could be a target for the development of novel intervention strategies against *Giardia*.

**Graphical abstract:**

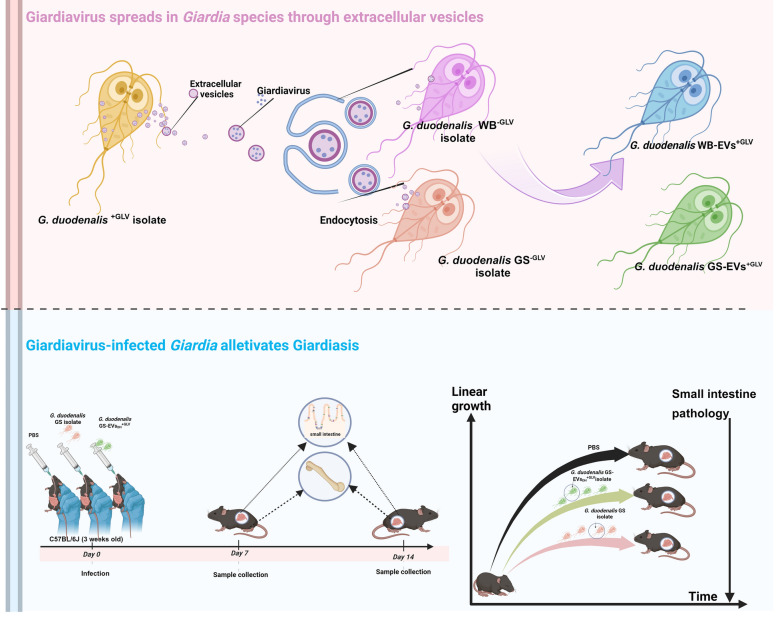

**Supplementary Information:**

The online version contains supplementary material available at 10.1186/s13071-025-06692-4.

## Background

*Giardia duodenalis* is the primary parasitic pathogen responsible for diarrheal disease in children and adult travelers, as well as childhood malnutrition and stunted growth in low- and middle-income countries (LMICs) [1, 2]. The *G*. *duodenalis* life cycle includes resistant cysts and proliferating trophozoites, which are ingested through cysts in contaminated water or food. Trophozoites colonize the lumen of the upper small intestine in vertebrates [1], attaching to the gut surface and causing the clinical manifestations of giardiasis. Additionally, *Giardia* infections may also cause some complications in certain situations, such as arthritis [3], postinfection irritable bowel syndrome (IBS) [4], allergies, impaired cognition, and myopathy [5, 6]. Research conducted by the Malnutrition and Enteric Diseases (MAL-ED) multicenter study demonstrated that asymptomatic *G*. *duodenalis* infection is an independent risk factor for impaired early-life linear growth in children from eight LMICs [7–9].

Extracellular vesicles (EVs) can transport viral proteins, DNA, and RNA to target cells [10], offer protection against neutralizing antibodies [11, 12], and mediate virus transmission [13], these highlight the significance of EVs in the viral life cycle. Reports have described two distinct types of EVs secreted by *Giardia* [14], and our group’s previous studies also found that *Giardia* secretes EVs [15]. Natali et al. revealed that the exosome-like vesicles of *Giardia* (derived from assemblages A, B, and E) contained distinct small RNA (sRNA) biotypes, and these sRNAs can be delivered from parasite to parasite via the EIVs [16]. Protozoan viruses specifically target protozoa. Giardiavirus (GLV), part of the Totiviridae family alongside other protozoan and fungal viruses [17], is the sole species in the genus *Giardiavirus*. It is a small, nonenveloped virus with a non-segmented double-stranded RNA (dsRNA) genome that encodes a capsid protein (ORF1) and an RNA-dependent RNA polymerase (ORF2). Studies have found that a positive rate of up to 36–47% for GLV in *G*. *duodenalis* isolates from human samples [18–20]. Given the common role of EV-mediated cell-to-cell and virus-to-cell communication, these insights provide new ideas for researching protozoan virus transmission among parasite species. However, it remains unreported whether the EVs transmission phenomenon applies to the GLV.

The presence of viruses within parasites alters the dynamics of the parasite–host relationship, adding complexity to the system by introducing a third partner. Protozoan viruses can cause serious human illnesses. LRV in *Leishmania* species from the Americas, and Trichomonasvirus (TVV) in *Trichomonas vaginalis*, can modify the virulence (degree of pathogenicity) of these parasites [20]. LRV1-infected parasites are responsible for exacerbating cutaneous leishmaniasis [21]. TVV infects other flagellated protozoa (*Trichomonas vaginalis*), affecting the severity of the disease caused in a human host [22]. However, the current knowledge about GLV infecting flagellated protozoans is still poor, and the influence of GLV infection on the virulence and pathogenicity of *Giardia* is also ambiguous. Only review articles have mentioned that GLV infection may cause the loss of intestinal adherence of the protozoan, resulting in a benign disease [23, 24], or that GLV-infected trophozoites exhibit similar fitness and pathogenicity compared with uninfected organisms [25]. Research is required to unravel the influence of GLV on the pathogenicity of *Giardia*. We explored in detail that partial GLV relies on the *G*. *duodenalis* exosomal pathway to transmit across parasites to establish GLV-infected *Giardia*, and EVs-enveloped GLV-infected *Giardia* alleviate the pathogenicity of *Giardia*. Our investigation has yielded a novel strategy for virotherapy from Giardiavirus, which may contribute to better-designed precautionary protocols and vaccines to provide protection against *Giardia* infection.

## Methods

### Parasites

The *Giardia* trophozoites utilized in our experiments were obtained as follows: GLV-free *Giardia* trophozoites originated from *G*. *duodenalis* WB Assemblage A1 (ATCC30957; American Type Culture Collection, Manassas, VA, USA). GLV-containing *Giardia* trophozoites were sourced from the parasite laboratory of the College of Veterinary Medicine, Jilin University, and were identified as the *G*. *duodenalis* Assemblage AI, named *Giardia*^+GLV^. Additionally, *G*. *duodenalis* GS/M trophozoites (50581^™^) were purchased from the American Type Culture Collection (ATCC). Trophozoites were cultured at 37 °C in modified TYI-S-33 medium supplemented with 12.5% heat-inactivated fetal bovine serum (Every Green, Zhejiang), 0.1% bovine bile (Sigma, USA), 50 mg/ml Gentamicin sulfate, 100 U/ml Penicillin, and 100 U/ml Streptomycin (Biological Industries, Israel). The tube was cooled on ice for 20 min, followed by centrifugation at 400 *g* for 8 min at 4 °C to harvest *G*. *duodenalis* trophozoites.

### Preparation of *Giardia* EVs and PKH67-labeled EVs

GLV-containing *Giardia* EVs isolation was conducted following protocols. Trophozoites were chilled on ice for 20 min to facilitate detachment from the tube, followed by two washes with phosphate buffered saline (PBS). EVs were collected from the supernatant of *Giardia*, with 1 × 10^6^/mL parasites incubated in TYI-S-33 medium with exosome-depleted serum at 37 °C for 12 h. The supernatant was collected and centrifuged at 400 *g* for 10 min to eliminate parasites, at 8500 *g* for 60 min to remove debris, followed by filtration through a 0.22 μm sterilized PES membrane (Merck Millipore, USA), and then further ultracentrifuged at 120,000 *g* for 1.5 h or using the exoEasy Kit (QIAGEN) [26–28]. The harvested EV was resuspended in 200 μL PBS. The exoEasy Maxi kit was used following the manufacturer’s protocol. In short, cell culture supernatant was added to an equal volume of “buffer XBP” and the mixture was centrifuged (500 *g*, 1 min, RT). Next, the filter was washed with “buffer XWP” and the EVs were eluted in 400 μL elution “buffer XE” (500 *g* for 5 min at RT, and then reapplied on the filter and centrifuged at 3000 *g* for 5 min at RT to maximize EV yield).

EVs were stained using the PKH67 Green Fluorescent Cell Linker Kit (Sigma-Aldrich, USA). In detail, 100 μL of PBS-EVs mixed with PKH67 (1:250 diluted with the Diluent C), the mixture was incubated for 4 min at RT in darkness, and then added to an equal volume of 0.5% bovine serum albumin (BSA) to remove excess dye. The PKH67-labeled EVs were washed with 10 mL PBS and collected by Amicon^®^ Ultra Filter, 100 kDa MWCO (Millipore).

### Scanning electron microscopy (SEM)

Trophozoites and duodenum samples were fixed in a 2.5% glutaraldehyde solution overnight at 4 °C. Subsequently, samples were placed on poly-l-lysine-coated slides and dehydrated using a series of ethanol, amyl acetate and supercritical CO_2_. The dehydrated specimens were then sputter-coated with Au–Pd and imaged using a HITACHI Regulus 8100 cold field emission gun scanning electron microscope (Servicebio, China).

### Transmission electron microscopy (TEM)

For negative staining, 10 μL of EVs were promptly applied to a carbon-coated copper grid and incubated for 1 min at room temperature (RT), followed by staining with 20 μL of 3% phosphotungstic acid for 5 min. Parasite pellets were initially fixed with 2.5% glutaraldehyde in 0.1 M sodium cacodylate buffer at RT for 30 min, and subsequently overnight at 4 ℃. After rinsing in the same buffer, the samples were post-fixed with osmium tetroxide, and dehydrated in a graded acetone series, and finally embedded in epoxy resin. Ultrathin sections (70–90 nm) were prepared from resin blocks using a Leica EM UC7 ultramicrotome. Formvar grids containing isolated vesicles or ultrathin sections were visualized using a HITACHI HT 7800 120kv transmission electron microscope.

### RNAse protection assay

EVs (100 µg) were subjected to different conditions, including no treatment, treatment with 1 µl of RNAse III (Ambion; 1Uμl − 1), in the presence or absence of 0.1% Triton X-100. The RNAse III reaction was carried out in 1 × RNAse III buffer (Ambion) at 37 ℃ for 20 min.

### Transfer assay

Donor parasites were introduced to the 0.4 µm pore-size inner chamber (Corning), while recipient parasites were placed in the wells. Additionally, an equal amount of EVs was added to the recipient parasite cultures in 22 ml culture bottle.

### Quantitative real-time PCR

Total RNA was extracted from *Giardia* trophozoites, EVs, duodenum, or liver using TRIzol reagent (TransGen Biotech), and the purity and concentration were validated by measuring the OD260 nm/OD280 nm ratio (1.8–2.0) using a Nanodrop ND-2000 spectrophotometer (Thermo Fisher Scientifc). Subsequently, DNA-free RNA (2 µg) was reverse transcribed with All-In-One 5X RT MasterMix (Abmgood) following the manufacturer’s protocol. Standardized amounts of cDNA and custom-designed primers were utilized for quantitative polymerase chain reaction (qPCR) or combined with BlasTaq^™^ 2 × qPCR MasterMix (SYBR Green, Abmgood) for gene transcription level analysis through qPCR assays conducted on a LightCycler 480 II platform (Roche Diagnostics GmbH, Mannheim, Germany). The relative mRNA change was determined using the 2-ΔΔCq method.

### Western blot analysis

*Giardia* EVs proteins were detected in line with the description [29]. Proteins were incubated with primary antibodies against HSP90, Rab 2a, and Rab 11 (polyclonal antibodies 1:200–1:1000, all of which are derived from mice and rabbits made in our laboratory).

### Nanoparticle Tracking Analysis (NTA)

The particle size and number of Giarida EVs were measured using Nanoparticle tracking analysis (NTA) with a ZetaView PMX 110 instrument (Particle Metrix, Germany). In detail, fresh EVs were diluted in PBS to a final volume of 1 mL, and their concentration was adjusted by observing a particles/frame rate of around 50 (30–100 particles/frame). For each measurement, five consecutive 60-s videos were recorded under the following conditions: cell temperature −25 °C, syringe speed-22 µL/s (100 a.u.). Captures and analysis were achieved by using the built-in ZetaView 8.02.28 software. the mean size, mode (i.e., the most represented EVs size population), and particles/mL were given by the software.

### Immunofluorescence assay

In total, 5 × 10^5^ *Giardia* trophozoites were adhered to L-lysine-coated glass slides for 30 min, then fixed with 4% paraformaldehyde for 20 min, followed by PBS/0.25% Triton X-100 for 10 min. After blocking in PBS/3% bovine serum albumin for 30 min, the cells were incubated with primary antibodies overnight at 4 °C and subsequently exposed to fluorescent dye-conjugated secondary antibodies. The samples were mounted using Antifade Mountant with DAPI (Beyotime, Shanghai, China) and examined by inverted confocal microscopy (model FV3000 microscope; OLYMPUS, Japan). The following antibodies were used at the specific dilutions: anti-dsRNA J2 mAb (1:100; English & Scientific Consulting Kft, Szirák), alexa fluor 488-conjugated goat anti-mouse immunoglobulin G (IgG) (1:200; proteintech).

### Animal studies

We used 3-week-old male C57BL/6 mice (weaned and wild-type), purchased from Liaoning Changsheng Biotechnology Co., Ltd. (Liaoning, China), that were acclimated to diets for 3 days pre-infection and continued on the same diets for 14 days postinfection. The mice were housed in a temperature-controlled environment (20 ± 2 °C) with a 12/12-h light/dark cycle, and provided ad libitum access to food and water. Mice underwent orogastric infection with a 100-μl inoculum containing 1.2 × 10^7^ *G*. *duodenalis* GS/M trophozoites in PBS. After infection, individual mouse weights were recorded serially until sacrifice. Euthanasia was confirmed via cervical dislocation before tissue collection.

### FITC–dextran uptake assay

Intestinal permeability was measured by the determination of FITC-dextran in serum after oral administration as described previously [8, 30]. Mice were subjected to a fasting period of ≥ 4 h before receiving 400 mg/kg FITC-dextran (4.4 kDa, Sigma-Aldrich, 46944) via intragastric gavage. Serum samples were collected after 4 h, and the FITC-dextran was determined using a multifunctional fluorescence microplate reader (M2e, Molecular Devices, Sunnyvale, CA, USA) with an excitation wavelength of 485 nm and an emission wavelength of 528 nm.

### Femur length measurement

Automatic detection and quantification of the femur were performed on x-ray computed tomography (CT) images. *Giardia*-infected mice at 7 and 14 days postinfection, were anesthetized using isoflurane-based respiratory anesthesia. A total-body CT scan was performed on the mice using a preclinical CT scanner with an x-ray tube voltage of 80 kV and power of 115 mA. The acquired three-dimensional (3D) CT images were reconstructed using RadiAnt DICOM Viewer for subsequent image analysis. Following the sacrifice of *G*. *duodenalis*–infected mice at 14 days postinfection, the femurs were extracted, fixed in 4% paraformaldehyde-PBS overnight at 4 °C, rinsed with PBS, and preserved in 70% ethanol. Bone length measurements were carried out using microCT (SkyScan 1276, Bruker, Germany), a technique of micro-computed tomographic imaging designed for small objects with pixel sizes in the micron or submicron range (1–100 μm). Reconstruction of the 3D image of the scanned object was achieved through the Feldkamp algorithm utilizing multiple x-ray transmission images obtained from incremental angular perspectives over 180° or 360°.

### Histopathology

Cross-sectional specimens of the duodenum, jejunum, and ileum from *Giardia*-infected and control mice were collected at 7 and 14 days postinfection, following the protocol outlined in reference [31]. Briefly, the tissue sections were fixed in 4% paraformaldehyde-PBS for 48 h and then transitioned to 70% ethanol. After fixation, the sections were embedded in paraffin, sectioned, and stained by the servicebio company. Photomicrographs were captured using an Olympus BX43 light microscope at magnifications of 40X and 100X with the assistance of cellSens Entry software.

### Statistical analysis

Statistical analyses were performed using the unpaired Student’s *t*-test (one- or two-tailed) or one-way analysis of variance. Error bars in the figures represent the standard error of the mean (SEM), with significance levels indicated as *P ≤ 0.05, **P ≤ 0.01, and ***P ≤ 0.001. Data analysis was carried out using GraphPad Prism software. The presented results are representative of a minimum of three independent experiments exhibiting consistent data.

## Results

### Partical Giardiavirus is secreted through the extracellular vesicles pathway

**Fig. 1:**
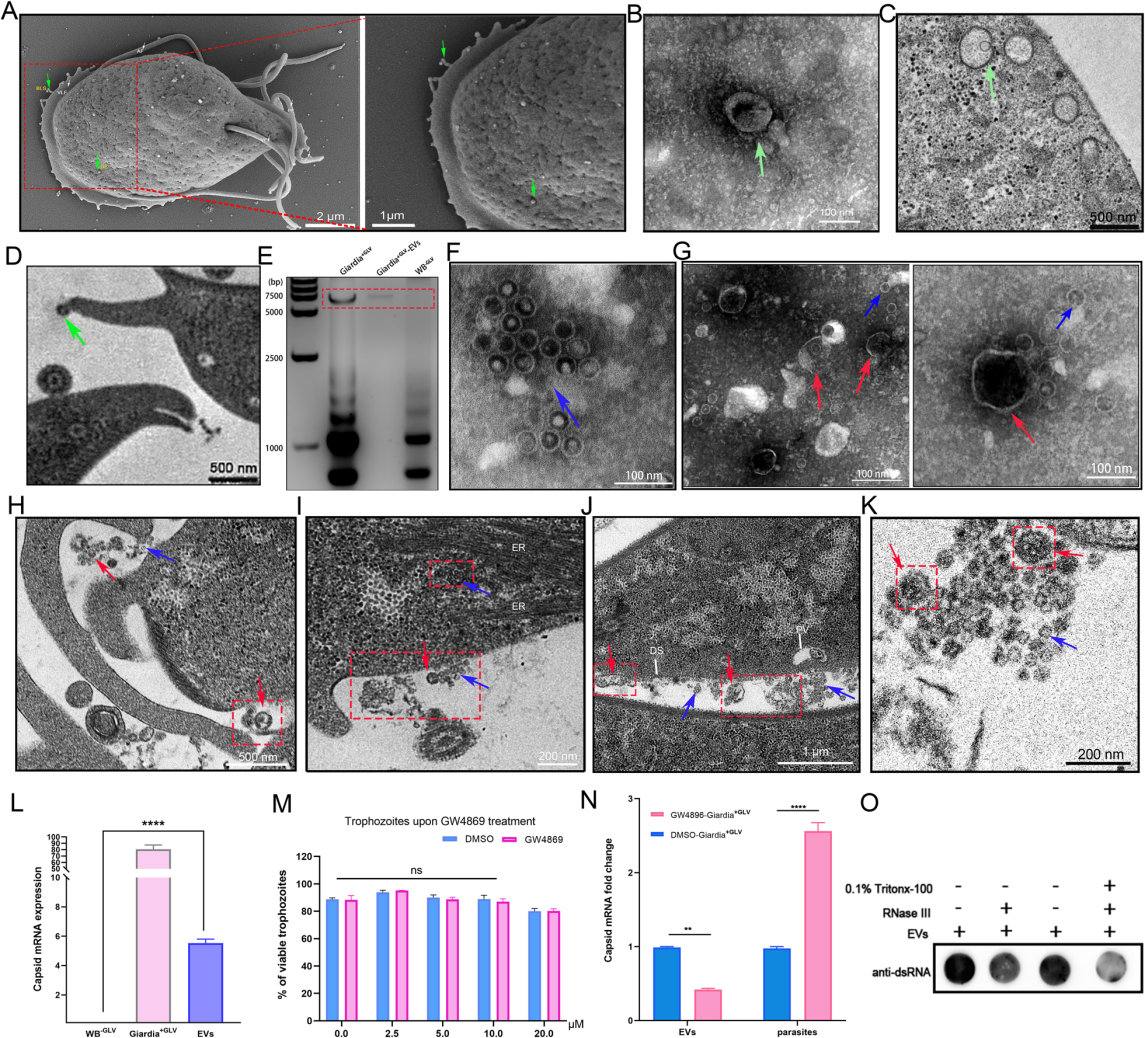
GLV virions exist in extracellular vesicles (EVs) of *Giardia* ^+GLV^ isolate. **A** Scanning electron microscopy observed EV secretion from *Giardia* ^+GLV^ trophozoites. **B** EVs derived from *Giardia* ^+GLV^ trophozoites were prepared for transmission electron microscopy (TEM) using negative staining. **C**, **D** *Giardia* ^+GLV^ was processed into resin slices and subjected to TEM observation. **C** Identification of EVs in peripheral vesicles in the *Giardia* ^+GLV^ isolate; **D** Localization of EVs at the ventrolateral flange. **E** The total RNA of *Giardia* ^+GLV^ trophozoites, EVs (derived from *Giardia* ^+GLV^) and WB^−GLV^ trophozoites were extracted and then analyzed by agarose gels. **F** Enrichment of EVs by ultracentrifugation, followed by preparation of GLV virions for TEM using negative staining. **G** GLV virions were encircled by vesicles for TEM using negative staining. **H**–**K** *Giardia* ^+GLV^ trophozoites were processed for TEM resin slices, GLV virions and EVs-enveloped GLV were observed. **L** Collection of *Giardia* ^+GLV^ EVs using exoEasy Maxi Kit and determination of GLV ORF1 (capsid) messenger RNA (mRNA) by qPCR, WB^−GLV^ trophozoites served as the negative control, and *Giardia* ^+GLV^ trophozoites served as the positive control. **M** *Giardia* ^+GLV^ trophozoites treated with the exosome inhibitor GW4869, cell viability assessed by trypan blue staining, and observation under an optical microscope. **N** Treatment of *Giardia* ^+GLV^ with the exosome inhibitor GW4869 for 12 h, followed by the collection of EVs and parasites to detect the level of GLV capsid. **O** RNase III is a class of endoribonucleases that is responsible for cleaving dsRNA, treatment of *Giardia* ^+GLV^ EVs with RNAse III in the presence or absence of Triton X-100, with detection of double-stranded RNA by dot blot. Green arrows indicate EVs, and the red box highlights important findings. Red arrows indicate membrane-enveloped GLV virions, green arrows represent GLV-free vesicles, and blue arrows indicate naked GLV virions. White letters label trophozoite structures

To investigate the extracellular vesicles (EVs) secreted by GLV-carrying *G*. *duodenalis*. GLV-containing *Giardia* trophozoites were genotyped by sequence analysis of the triosephosphate isomerase (TPI) and β-giardin genes [32, 33], identifying them as Assemblage AI, named *Giardia*^+GLV^ (Supplementary Fig. 1). We found that the *Giardia*^+GLV^ isolate secreted numerous vesicle-like structures. SEM showed that these vesicles were clustered on the *Giardia* surface, with free vesicles observed on the dorsal side (DS) and bead-like structures (BLS) on the ventrolateral flange (Fig. 1A, Supplementary Fig. 2A-B). Vesicles from *Giardia*^+GLV^ trophozoites were observed by TEM negative staining, showing that vesicles have a typical spherical or cup-shaped membrane bilayer with a diameter of 100–200 nm (Fig. 1B). The proteins involved in EVs formation were detected in the EVs of *Giardia*^+GLV^ by proteomic and western blotting analysis, incuding Rab 2a, Rab 11, and HSP 90 (Supplementary Fig. 3A). NTA also revealed that *Giardia*^+GLV^ EVs exhibited a mean diameter of 134.4 nm and a concentration of 1.9 × 10^10^ particles/mL (Supplementary Fig. 3B). Additionally, we observed that vesicles existed at peripheral vesicles (PVs) (Fig. 1C, Supplementary Figs. 3C-F), and bead-like vesicles existed at the ventrolateral flange (Fig. 1D, Supplementary Fig. 3G-J).

Next, we further investigate whether the GLV can be released into the extracellular space through EVs. EVs obtained by ultracentrifugation also contained GLV dsRNA (Fig. 1E). We also employed TEM to observe GLV virions and aimed to establish whether GLV was packaged within *Giardia* EVs. TEM analysis showed that GLV virions accumulated in the extracellular environment (Fig. 1F), were present in EVs surrounding, and suspected GLV-containing vesicles were observed (Fig. 1G). The release of GLV virions, multiple vesicles and membrane-enveloped GLV-likeparticles in close proximity to the parasite plasma membrane was observed (Fig. 1H–K). Additionally, GLV signals were also detected near the endoplasmic reticulum (ER) (Fig. 1I). GLV virions were collected for whole genome sequencing, which provides full viral genomic information for study of the virus, we uploaded the GLV information to the NCBI database (PQ212523, Supplementary Fig. 4). To rule out that GLV-containing EVs were an artifact from the multiple centrifugation steps, we utilized an exoEasy Maxi Kit to isolate GLV-containing *G*. *duodenalis*-secreted EVs. The GLV ORF1 (capsid) was detected in EVs through qPCR (*P* < 0.001, Fig. 1L). *Giardia* trophozoites were treated with the exosome inhibitor GW4869 to reduce EVs. Cell viability was assessed by trypan blue staining, and 10 μM of GW4869 was used for subsequent experiments (*P* > 0.05, Fig. 1M). Trophozoites were treated with GW4869, EVs showed a decrease in capsid levels (*P* < 0.01), while trophozoites showed an increase (*P* < 0.001)(Fig. 1N, Supplementary Fig. 5D). Additionally, *Giardia*^+GLV^ EVs were treated with RNase III in the presence or absence of 0.1% Triton X-100, followed by detection of viral dsRNA using Dot blot (Fig. 1O) and semi-quantitative PCR (Supplementary Fig. 5E). RNA protection assays demonstrated that the EVs coating shielded the viral dsRNA genome from degradation by RNase III, confirming the presence of viral dsRNA in EVs (Fig. 1O). Overall, our investigations showed the presence of GLV particles in *Giardia*-secreted EVs.

### GLV Spreads among *Giardia* species via EVs

**Fig. 2:**
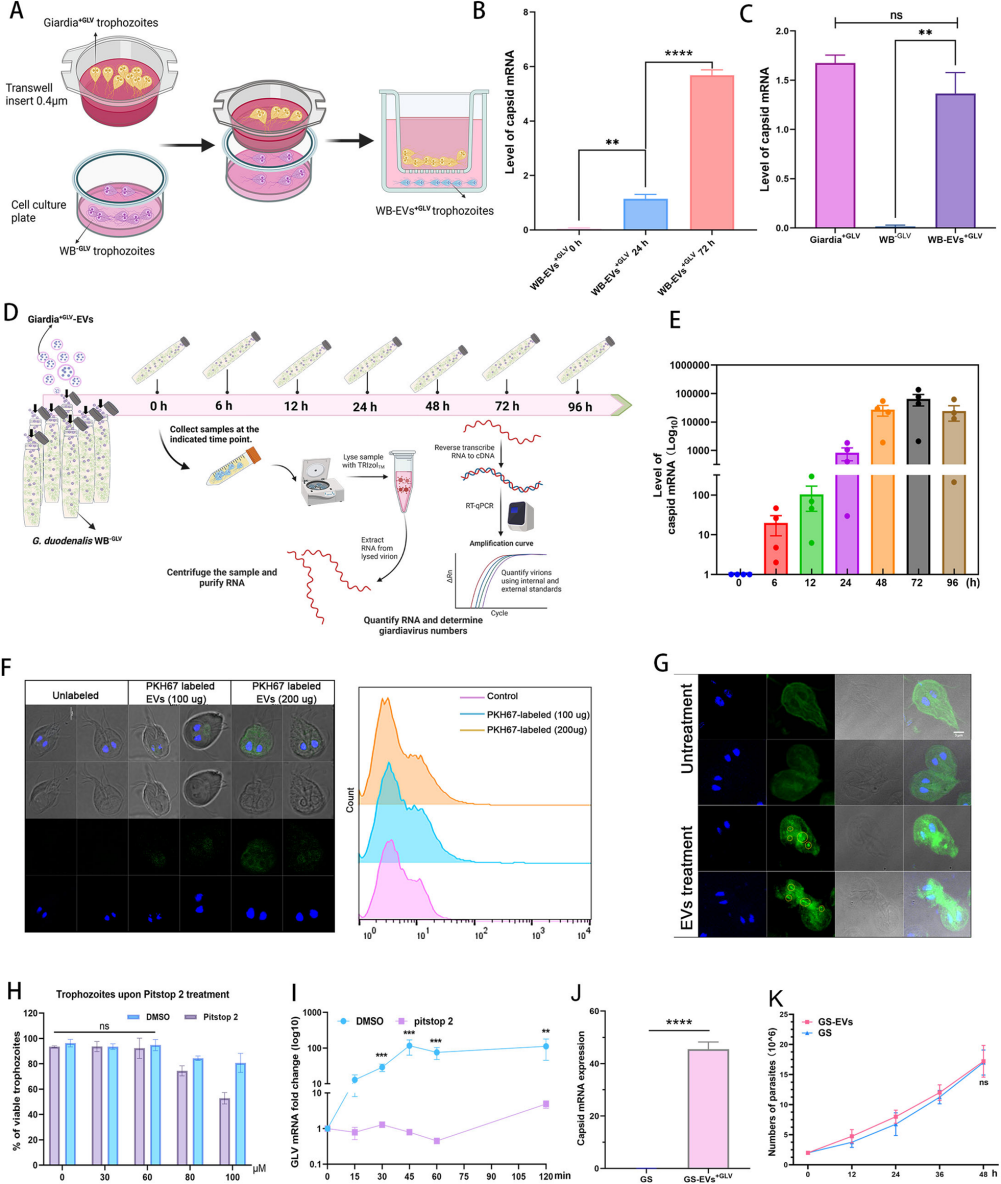
EVs encapsulation of GLV: a route of transmission for GLV among *Giardia* species. **A** The transwell migration settings of artificially constructed GLV-infected WB^−GLV^ strain, *Giardia*^+GLV^ trophozoites added to the 0.4 µm pore-size chamber and WB^−GLV^ trophozoites added to the wells. Trophozoites were collected at the indicated time points (**B**) or at 2 weeks postinfection **C** for total RNA extraction, the detection of GLV ORF1 (capsid) by qPCR. **D** The equal amount of EVs (collected from the exoEasy Maxi Kit) from the same source were introduced into *G*. *duodenalis* WB^−GLV^ cultures (2 × 10^^^6 parasites) in a 22 ml tube. **E** Trophozoites were collected at various time points (0 h, 6 h, 12 h, 24 h, 48 h, 72 h, and 96 h) for total RNA extraction, followed by the detection of capsid by qPCR. **F** *Giardia*^+GLV^ EVs were labeled with PKH67, and added to WB^−GLV^ cultures, the uptake of EVs was measured by confocal microscopy and flow cytometry after co-incubation. Scale bar, 5 μm. **G** *Giardia*^+GLV^ EVs were added to WB^−GLV^ cultures, and GLV double-stranded RNA was detected by immunofluorescence. Scale bar, 3 μm. **H** WB^−GLV^ trophozoites were treated with various concentrations (20–100 μM) of pitstop2 for 24 h to inhibit endocytosis. Trophozoites treated with an equal dose of dimethyl sulfoxide (DMSO) served as the control, and the viability of trophozoites was then assessed. **I** WB^−GLV^ were pretreated with 60 μM pitstop 2 (added 2.8μL of 150 mM into 7 mL) or DMSO (2.8 μL), followed by WB^−GLV^ infected with EVs, artificially constructed the GLV-infected *Giardia* strain was collected at various time points for capsid detection. **J** *G*. *duodenalis* GS trophozoites infected with GLV via *Giardia*^+GLV^ EVs to construct GS-EVs^+GLV^ trophozoites, the capsid level was detected in GS-EVs^+GLV^ trophozoites by qPCR. **K** The growth rate of GS trophozoites infected with and without GLV was measured

Protozoan parasites utilize EVs to mediate cell–cell communication during host-parasite interactions [34]. We investigated the potential transport of viral particles between *Giardia* species via EVs. Transwell migration experiments were conducted using *Giardia* ^+GLV^ trophozoites as donor cells and *G*. *duodenalis* WB^−GLV^ trophozoites as recipient cells (Fig. 2A). After co-incubation for 24 h, *Giardia* ^+GLV^ trophozoites were removed, WB^−GLV^ recipient cells were cultivated for up to 72 h, qPCR showed that the level of GLV capsid was increased (*P* < 0.01, Fig. 2B). To ascertain whether the GLV is persistently sustained in the WB^−GLV^ strain, *Giardia* ^+GLV^ trophozoites were removed after co-incubation for 24 h, and WB^−GLV^ trophozoites were transferred to fresh culture media and cultured for 2 weeks, the GLV capsid level was still detected and sustained in trophozoites (*P* < 0.01, Fig. 2C), allowing for the establishment of GLV-infected parasite lines, designated WB-EVs^+GLV^(artificially constructed GLV-infected *Giardia* strain). To further elucidate whether EV-enveloped GLV serves as one of the transmission routes for GLV in inter-species transfer among *G*. *duodenalis*, we introduced EVs collected from the exoEasy Maxi Kit to *G*. *duodenalis* WB^−GLV^ cultures (Fig. 2D). Our findings showed a time-dependent trend in GLV capsid levels in recipient cells during the 0–72 h incubation period, with a subsequent decrease observed at 96 h compared with 72 h (Fig. 2E).

To visualize the uptake of EVs by *G*. *duodenalis* WB^−GLV^ species, we employed green fluorescent dye PKH67-labeled EVs and examined them using a confocal microscope and flow cytometry. Our analysis revealed the fluorescence signals in WB^−GLV^ recipient cells (Fig. 2F). Upon the addition of *Giardia* ^+GLV^-derived EVs to WB^−GLV^ cultures, a more pronounced green fluorescence, immunostained with the anti-dsRNA J2 mAb was evident in the trophozoites infected with GLV (Fig. 2G). We also applied pitstop 2 to pretreat *Giarida* WB^−GLV^, an inhibitor of clathrin-dependent/independent endocytosis, to block EVs uptake via the endocytic pathway, and assessed its impact on the entry of EVs-encapsulated GLVs. We found that Pitstop 2, at a concentration of 60 µM, had no toxic effects on WB^−GLV^ trophozoites (*P* > 0.05, Fig. 2H). The uptake of EVs-coated GLV by the WB^−GLV^ isolate was observed as early as 15 min, peaking at 45 min. In contrast, the pitstop 2 treatment group had lower, delayed, and non-transient uptake, with no tendency for elevated viral load over 120 min (*P* < 0.01, Fig. 2I), preliminary evidence indicates that GLV-encapsulating EVs enter *Giardia*^−GLV^ via the endocytosis pathway. Next, we added EVs^+GLV^ collected from the exoEasy Maxi Kit to *G*. *duodenalis* GS^−GLV^ cultures, following the same procedure as described above, GLV-infected GS strain (GS-EVs^+GLV^) was constructed and showed sustained GLV infection (*P* < 0.001, Fig. 2J). The growth of the GS isolate with and without GLV was monitored, there was no significant difference between the GS-EVs^+GLV^ strain and the GS isolate (*P* > 0.05, Fig. 2K). Overall, EVs-enveloped GLV serves as one of the transmission routes for GLV in inter-species transfer among *Giardia* species.

### GLV infection attenuates growth restriction caused by *Giardia*

**Fig. 3:**
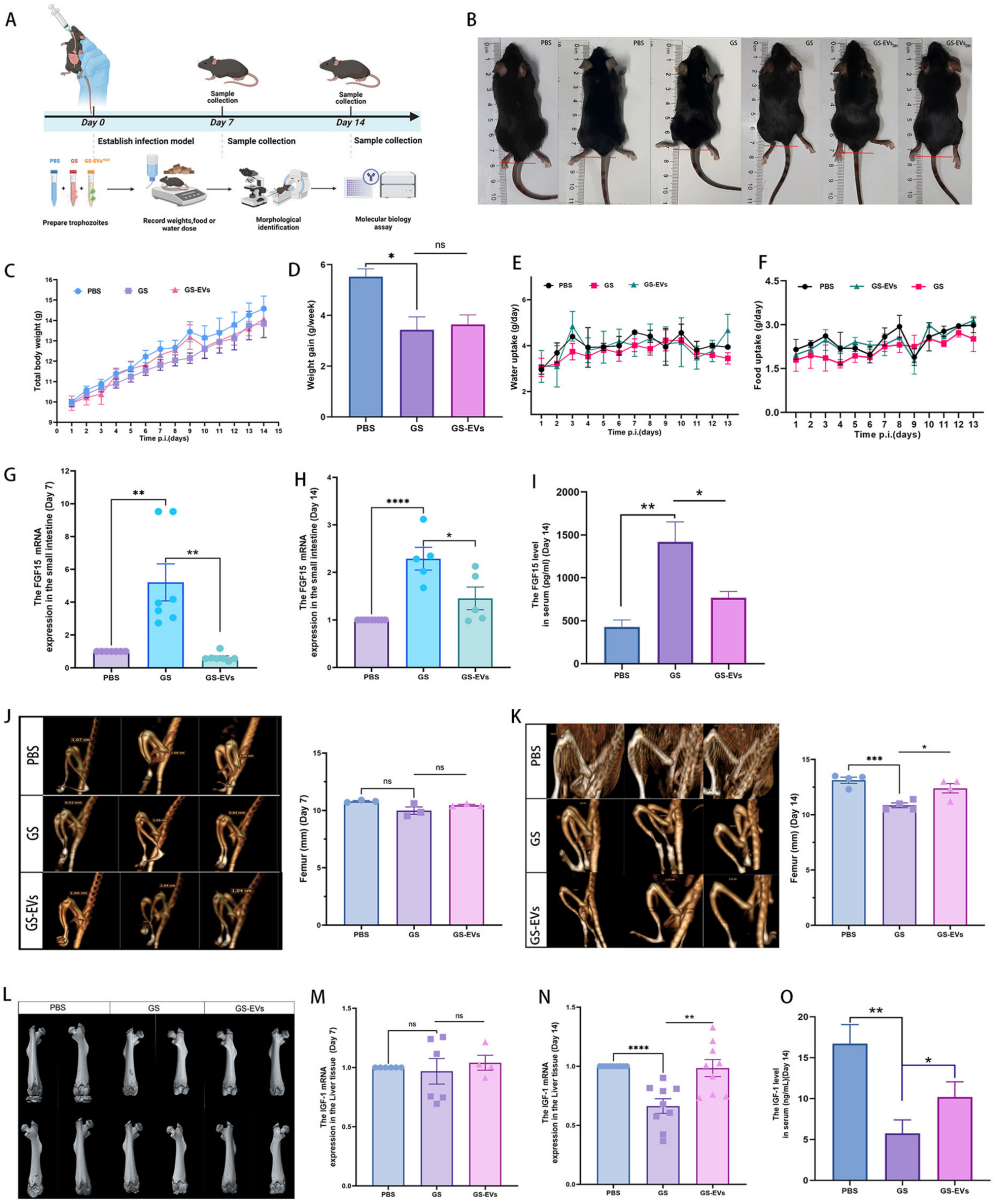
GLV-infected *Giardia* alleviates symptoms of giardiasis. **A** An overview of the experimental setup. **B**–**D** *G*. *duodenalis* GS trophozoites and the GS-EVs^+GLV^ trophozoites were orally administered to mice, and the macroscopic growth parameters (weight and size gains) of C57BL/6J male mice were monitored for up to 2 weeks postinfection. **E**, **F** Daily recording of water and food intake by the mice. **G**, **H** FGF15 mRNA expression in the small intestine was quantified by qPCR on days 7 and 14 postinfection. **I** The level of FGF15 protein in serum was examined by ELISA kit at the 14th postinfection. **J**, **K** The femur bone length of the mice was evaluated using electronic computer X-ray tomography (μCT) at the 7th and 14th days postinfection. **L** MicroCT statistical analysis of femur length in mice. Three-dimensional (3D) volume renderings of reconstructed femur bone showing the front and back of each sample at the 14th day postinfection. **M**, **N** Analysis of liver insulin-like growth factor-1 (IGF-1) expression by qPCR at the 7th and 14th days postinfection. **O** The level of IGF-1 protein in serum was examined by ELISA kit at the 14th day postinfection

To investigate the effect of GLV infection on illness symptoms in mice caused by *Giardia*, we orally administered the GS isolate and the GS-EVs^+GLV^ strain to 3-week-old C57 mice, establishing *Giardia* GS or GS-EVs^+GLV^-infected mouse models (Fig. 3A, Supplementary Figs. 6A-B). Assessing macroscopic growth parameters (weight and size gains) in C57 mice fed a maintenance diet for 2 weeks, the results revealed that *Giardia* GS-EVs^+GLV^-infected mice exhibited stunted growth and reduced body size, which were alleviated compared with GS-infected mice (Fig. 3B). The diminished length gain was accompanied by lower total weight gain (Fig. 3C, D), and decreased final water and feed intake (Fig. 3E, F), while these symptoms were relieved in mice infected with GS-EVs^+GLV^ strain (*P* > 0.05). To further investigate the emaciation symptoms induced by *Giardia* infection at the molecular level. Fibroblast growth factor 15 (FGF15) levels in the small intestine and serum of mice were detected. FGF15 was significantly increased in GS-infected mice compared with the other two groups at 7 and 14 days postinfection, GS-EVs^+GLV^-infected mice showed an increase only at 14 days postinfection, with levels significantly lower than those in GS-infected mice (*P* < 0.01, Fig. 3G, H). The level of FGF15 protein in serum at 14th day postinfection showed the same trend (*P* < 0.05, Fig. 3I). Femur length is a bone measurement used in growth assessments. On day 14 postinfection, we noted that the femur length of mice orally gavaged with GS was shorter compared with the other two groups, but it was significantly reversed in the GS-EVs^+GLV^ group (*P* < 0.05). These symptoms were also observed on day 7 postinfection, though no significant difference was found (Fig. 3J–L). Additionally, lower levels of insulin-like growth factor-1 (IGF-1) were associated with the impairment in body growth and poor nutrition. Molecular analysis revealed a decrease in IGF-1 mRNA levels in the liver and protein levels in the serum of GS-infected animals on day 14 postinfection, but these symptoms were reversed in the GS-EVs^+GLV^ group (*P* < 0.05, Fig. 3N, O). No significant changes in IGF-1 were observed among the three groups on day 7 postinfection (Fig. 3M). Our findings suggest that the malignant effect of *Giardia* on linear growth in mice is attenuated in GLV-infected *Giardia*.

### GLV-infected *Giardia* decreases *Giardia*-mediated gut dysfunction

**Fig. 4:**
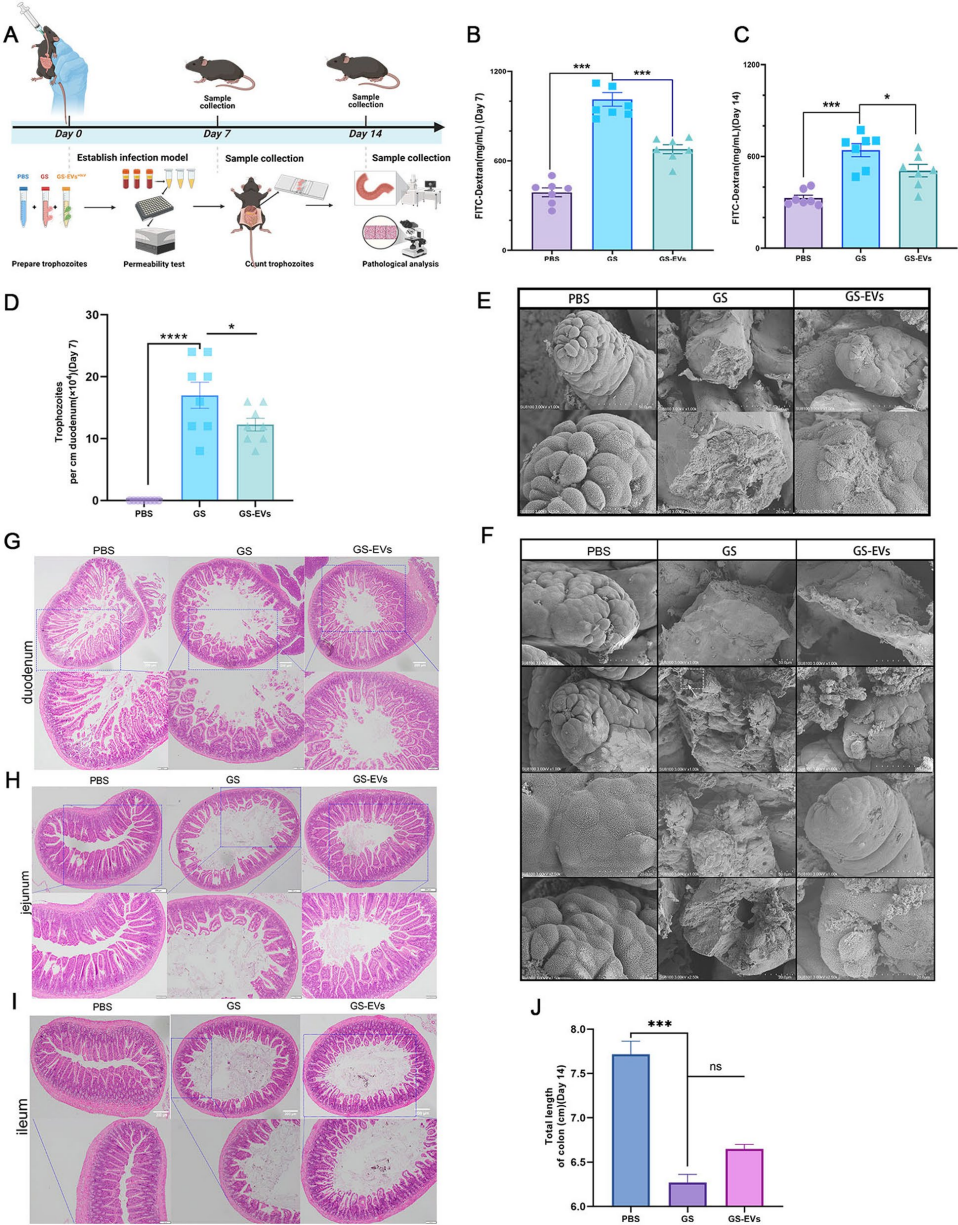
Reduced intestinal damage in *Giardia GS*-EVs^+GLV^-infected mice. **A** An overview of the experimental setup. **B**, **C** *G*. *duodenalis* GS wild-type trophozoites and GS-EVs^+GLV^ trophozoites were orally administered to mice, intestinal permeability was assessed using FITC-labeled Dextran reagent on the 7th (**B**) and 14th (**C**) days postinfection. **D** Counts of trophozoites on per cm of duodenum tissue in mice infected with *Giardia*, at day 7 postinfection. **E**, **F** Morphological examination of the proximal duodenum of mice was conducted using scanning electron microscopy. **G**–**I** Hematoxylin–eosin staining of the mouse duodenum, jejunum, and ileum at the 7th day postinfection. Original magnification, × 4, and the dashed blue squares indicate enlarged regions, magnification × 10. **J** Measurement of the colon length of mice on day 14 postinfection

To investigate the host’s intestinal mucosal response to *Giardia* GS infected with or without GLV. FITC-dextran was gavaged into mice to analyze intestinal permeability (Fig. 4A). Our results showed that intestinal permeability was significantly increased in mice infected with *G*. *duodenalis*. However, intestinal permeability in GS-EVs^+GLV^-infected mice was restored, with levels significantly lower than in GS-infected mice on the 7th (*P* < 0.001) and 14th (*P* < 0.05) days postinfection (Fig. 4B, C). On the 7th day postinfection, trophozoites were detected in the luminal contents of the proximal part of the small intestine in *Giardia*-infected mice, and the results found that more trophozoites exsited in GS-infected mice than in GS-EVs^+GLV^-infected mice (Fig. 4D). Ultrastructural analysis revealed that the duodenum barrier destruction in *Giardia* GS-infected mice was severe, with damage to the mucous layer, villi detachment, shedding of epithelial cells, and central lacteal exposure. In contrast, mice infected with GS-EVs^+GLV^ trophozoites exhibited much milder intestinal symptoms (Fig. 4E, F). Histopathology showed that *G*. *duodenalis* induced extremely severe lesions in the small intestine, including the duodenum, jejunum and ileum, where villi atrophy, shortening of intestinal villi, and inflammatory cell infiltration were clearly observed. Conversely, mild histopathologic symptoms were observed in the GS-EVs^+GLV^ group (Fig. 4G–I). Additionally, a shorter colon was observed in *Giardia* GS-infected mice compared with GS-EVs^+GLV^-infected mice (*P* > 0.05, Fig. 4J, Supplementary Fig. 6C). Our findings suggest that *Giardia* GS infected with GLV derived from the *Giardia*^+GLV^ EVs poses less risk to the intestine of mice.

## Discussion

Extracellular vesicle (EV)-mediated signaling within the virus–host interaction network is an emerging field of research [35], and is extensively involved in the replication, transmission, and infection of various viruses [36]. Giardiavirus is a unique virus that infects *G*. *duodenalis*, and has the ability to release virions into the culture medium. It has also been reported that GLV virions exhibit greater thermos-resistance compared with other species [17]. However, it remains unclear whether GLV, like most other members of the Totiviridae family, such as Leishmaniavirus and Trichomonas vaginalis virus, can be encapsulated in EVs and released into the environment [37–39]. EVs secretion by GLV-free *Giardia* isolates has been widely reported [15, 27]. We observed that bead-like structures (BLS) also secreted on the ventrolateral flange of *Giardia* WB^−GLV^ trophozoites (Supplementary Fig. 2C-D), and these BLS resemble the tunneling nanotube-like structures in the *Giardia* WB isolate reported by Midlej et al., but we lack sufficient evidence to confirm they are the same structure [40, 41]. EVs form when multivesicular bodies (MVBs) fuse with the plasma membrane and release the intraluminal vesicles (ILVs) [42]. *G*. *duodenalis* trophozoites possess a specialized organelle known as peripheral vesicles (PVs), composed of a group of approximately 150 nm vesicles distributed beneath the plasma membrane on the dorsal side and in a specific region near the ventral disc [43]. Sofía Moyano et al. have indicated the presence of intraluminal vesicles (ILV) within some endosome/lysosome peripheral vacuoles (PVs) of *G*. *duodenalis* species [44], suggesting a potential secretory function of PVs possibly linked to MVBs [45, 46]. Despite the absence of classical MVBs, exosome-like vesicles were formated within PVs [16, 47], our findings also support these.

The first question we addressed was whether GLV could be released into the environment via EVs. Although previous research has shown aggregates of GLV particles are commonly present outside cells, often associated with microvesicles (MVs), no further research has been explored [18]. In our experiments, GLV enveloped within the EV was revealed by TEM, two distinct populations of GLV-containing particles: one is EVs-enveloped GLV, and the other is the naked GLV virions. These observations align with previous reports of viral particle clusters, including LRV1 and hepatitis A virus [37, 48]. We also further isolated EVs using the standard membrane affinity spin column approach (EVs collected by the exoEasy maxi kit) [49], a method previously used to collect EVs deveried from *G*. *duodenalis* [28]. GLV, a non-enveloped virus without a phospholipid membrane component [50], is unable to bind to the membrane affinity rotating column, and the capsid was detected in EVs collected from the exoEasy maxi kit. Notably, this method successfully excluded the contamination of small vesicles (similar in size to the GLV particle) that can occur with cesium chloride gradient centrifugation. Duarte et al. identified several predominant neutral sphingolipids in *Giardia* [51, 52], so the nSMase inhibitor GW4869 was employed to decrease EVs release [53]. We observed that the capsid level was also reduced by the exosome inhibitor GW4869 treatment. These findings provide further evidence that GLV can be released via EVs derived from *G*. *duodenalis*.

The second question we explored was whether GLV transfers among *Giardia* species via EVs. Although a 1993 study reported that GLV virions enter susceptible WB trophozoites via endocytosis [54]. In recent years, researchers have discovered that the virus-carrying EVs (which transfer the virus and other components) play an important role in virus transmission [35]. Various viruses, including human immunodeficiency virus (HIV) [55] and Epstein–Barr virus [56], utilize host exosome/EVs machinery for their formation, spread, intercellular communication, and modulation of host responses. The *Totiviridae* family in protozoa includes three genera: Giardiavirus (GLV), Leishmaniavirus (LRV), and Trichomonasvirus (TVV), all sharing common characteristics [22]. EV-mediated transmission of TVV and LRV, which facilitates virus transmission among parasites [37–39]. Our results showed that GLV-carrying EVs (*Giardia*^+GLV^ EVs collected by the exoEasy maxi kit) were introduced into WB^−GLV^ isolates, which were successfully infected with GLV and maintained a persistent infection. GLV infection typically does not harm host cells, but there are also reports that the accumulation of a high viral load leads to the cessation of *G*. *duodenalis* trophozoites division and cellular function, resulting in parasite growth arrest [18, 57]. In our experiment, Fig. 2E shows a decrease in capsid levels at 96 h compared with 72 h, we speculate that this might be related to the phenomenon we observed, which was that GLV-carrying EVs infected GLV-free *Giardia*, causing *Giardia* to gradually die after the third day of infection, with this situation persisting for about 3 days. Afterwards, GLV and *Giardia* seem to establish a balanced relationship and proliferate steadily. These observations suggest that certain mechanisms exist in *Giardia* that regulate the balance between growth of *Giardia* and GLV, but further exploration is needed in the future.

Confocal microscope and flow cytometry to examine green fluorescent dye PKH67-labeled EVs in *G*. *duodenalis* WB^−GLV^ species [28, 37]. Consistent results were obtained when parasites were immunostained with the *Giardia* capsid and the anti-dsRNA J2 mAb [58, 59]. Therefore, we labeled the GLV dsRNA in *Giardia* WB-EVs^+GLV^ using dsRNA J2 mAb and observed an increase in green fluorescence. These findings suggest that EVs transferred GLV into GLV-free *Giardia* trophozoites. Additionally, we also found that the EV coating serves as a protective shield for GLV against RNase III, suggesting that EVs-coated GLV may act as a defense mechanism, and this mechanism may evolve to shield the virus from extracellular threats, such as degrading enzymes, evading neutralizing antibodies [48], or the host immune system [60]. EVs are primarily taken up by recipient cells via endocytosis [61, 62], *G*. *duodenalis* has developed endocytic mechanisms for the uptake of molecules from the extracellular environment [63, 64]. Studies have explored various inhibitors to treat *Giardia* and investigate different experimental approaches [65, 66]. Therefore, *Giardia* WB^−GLV^ were pretreated with the clathrin-dependent/independent endocytosis inhibitor pitstop 2 [67], GLV infection was also delayed, and the capsid level of WB-EVs^+GLV^ was decreased, these results tentatively reveal that the GLV-carrying EVs from *Giardia*^+GLV^ enter trophozoites via endocytic pathways.

We introduced EVs (*Giardia*^+GLV^ EVs collected by the exoEasy maxi kit) into the GS^−GLV^ isolates, which were successfully constructed into the GS-EVs^+GLV^ strain. Establishing an EVs-mediated GLV transmission model is more conducive to exploring the impact of GLV on illness symptoms caused by *G*. *duodenalis* to eliminate factors of different strains. The presence of viruses within parasites alters the dynamics of the parasite-host relationship, adding complexity to the system by introducing a third partner. Protozoan virus-infected parasites can cause the worsening of human illnesses, such as LRV and TVV-infected parasites [21, 22]. However, our results showed that GLV-infected *Giardia* mitigated the progression of giardiasis. Previous studies have indicated notable weight loss and impaired growth in *Giardia*-infected individuals [8, 68]. *G*. *duodenalis* is also associated with disruption of the intestinal barrier [9]. Our results demonstrate the restoration of growth inhibition and intestinal damage induced by GLV-infected *Giardia* through morphological and molecular analysis. FGF15 plays a role in regulating lipid oxidation and energy expenditure in adipocytes, impacting body adipose tissue accumulation [68], thereby providing additional evidence of emaciation symptoms in mice infected with *Giardia.* Analysis of FGF15 levels in mice infected with the GS-EVs^+GLV^ strain was lower than in mice infected with the GS strain, further confirming the attenuation of weight loss and emaciation. These findings also revealed that the diminished weight gain in *Giardia*-infected animals was not attributable to reduced food intake.

Additionally, lower levels of insulin-like growth factor-1 (IGF-1) are associated with impaired body growth and poor nutrition [30, 69]. The reversal in insulin-like growth factor-1 (IGF-1) production in mice infected with the GS-EVs^+GLV^ strain also confirmed the alleviation of macroscopic growth parameters (weight and size gains) and femur length. Intestinal permeability is an important indicator for assessing gut health, covering aspects, such as structural integrity, mucosal barrier function, and permeability to substances [70], and intestinal permeability is also associated with percent growth [8]. *G*. *duodenalis* infection disrupts epithelial permeability, enabling gut bacteria to invade. Loss of intestinal barrier function is a key contributor to the acute and post-infectious complications of *Giardia* infection [71]. Reduced intestinal damage induced by *Giardia* GS-EVs^+GLV^ was also found in our results, including in intestinal permeability and pathology. Although we have found that GLV-infected *Giardia* reduced symptoms in mice, GLV-infected *Giardia* might influence gut microbiota changes in the host intestinal flora, leading to different symptoms rather than directly affecting *Giardia* itself. We will further investigate the effects of GLV infection on the virulence and pathogenicity of *Giardia*, such as by comparing the protein composition of *Giardia* after GLV infection (including pathogenic proteins) or examining differences in the modulation of host mechanisms.

## Conclusions

*Giardia* EVs act as mediators that can mediate parts of the GLV transmission and provide protection for the GLV. Notably, the GLV-infected GS strain caused attenuated lesions in mice compared with GS wild-type isolates, revealing that GLV infection reduces the lesions caused by *Giardia*. These findings also suggest that GLV has potential as a target for the development of novel intervention strategies against *Giardia* infections.

## Supplementary Information

Supplementary material 1.

## Data Availability

The authors declare that the dataset has been deposited in the ProteomeXchange (PXD054439) and NCBI database (PQ212523), or are also available from the authors upon request.

## Abbreviations

PV: Peripheral vesicles
IC: Intracellular compartments
VAD: Ventral adhesive disc
DS: Dorsal side
ER: Endoplasmic reticulum

## Key structures of *Giardia *^+GLV^ trophozoites are labeled as follows:

AF: Anterior flagellum
PF: Posterolateral flagella
VF: Ventral flagella
CF: Caudal flagella
VLF: Ventrolateral flange
DS: Dorsal side
BLS: Bead-like structures
