# Supplementary material for: Giardiavirus infection alleviates growth restriction and intestinal damage caused by the intestinal parasite Giardia duodenalis
Source: Parasit Vectors. 2025 Feb 24;18:71. doi: 10.1186/s13071-025-06692-4 (PMC11853539; doi:10.1186/s13071-025-06692-4)
Supplement: Supplementary file 1 — Supplementary material 1. [file 13071_2025_6692_MOESM1_ESM.docx]

**Supplementary materials**

**Examination of Giardiavirus-containing *Giardia* isolate genoty**pes


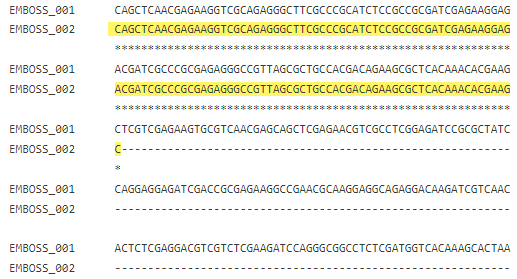

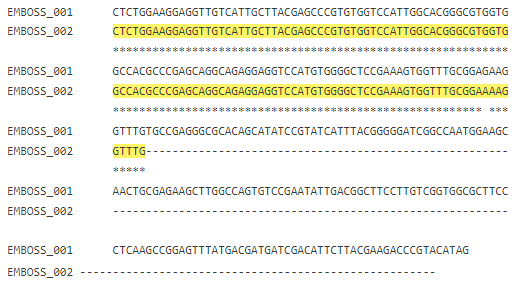

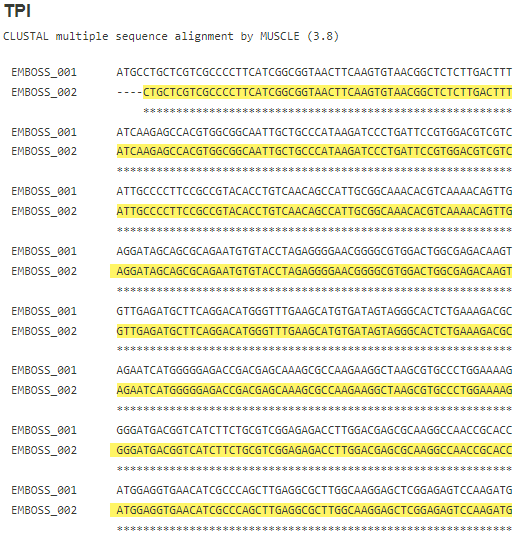

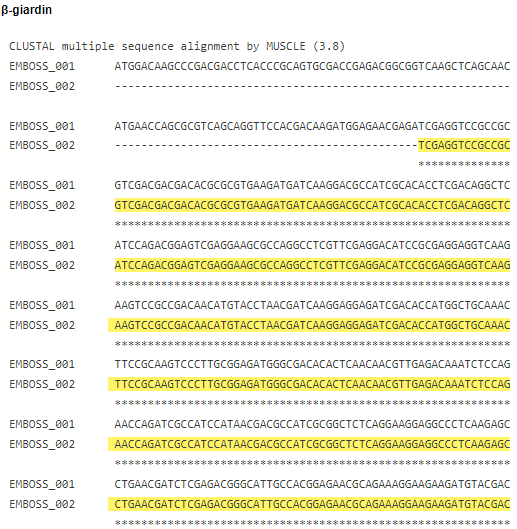


**Supplementary Fig. 1** The subgenotypes identification of GLV-containing *Giardia* trophozoites.

Partial b-giardin nucleotide sequences of GLV-containing *G. duodenalis* trophozoites were aligned, with the Portland-1 strain (GenBank accession number X14185) serving as the reference. GenBank accession number L02120 was chosen as the reference for the alignment of partial TPI nucleotide sequences of GLV-containing *G. duodenalis* trophozoites.

***Giardia*^+GLV^ trophozoites secrete extracellular vesicles**


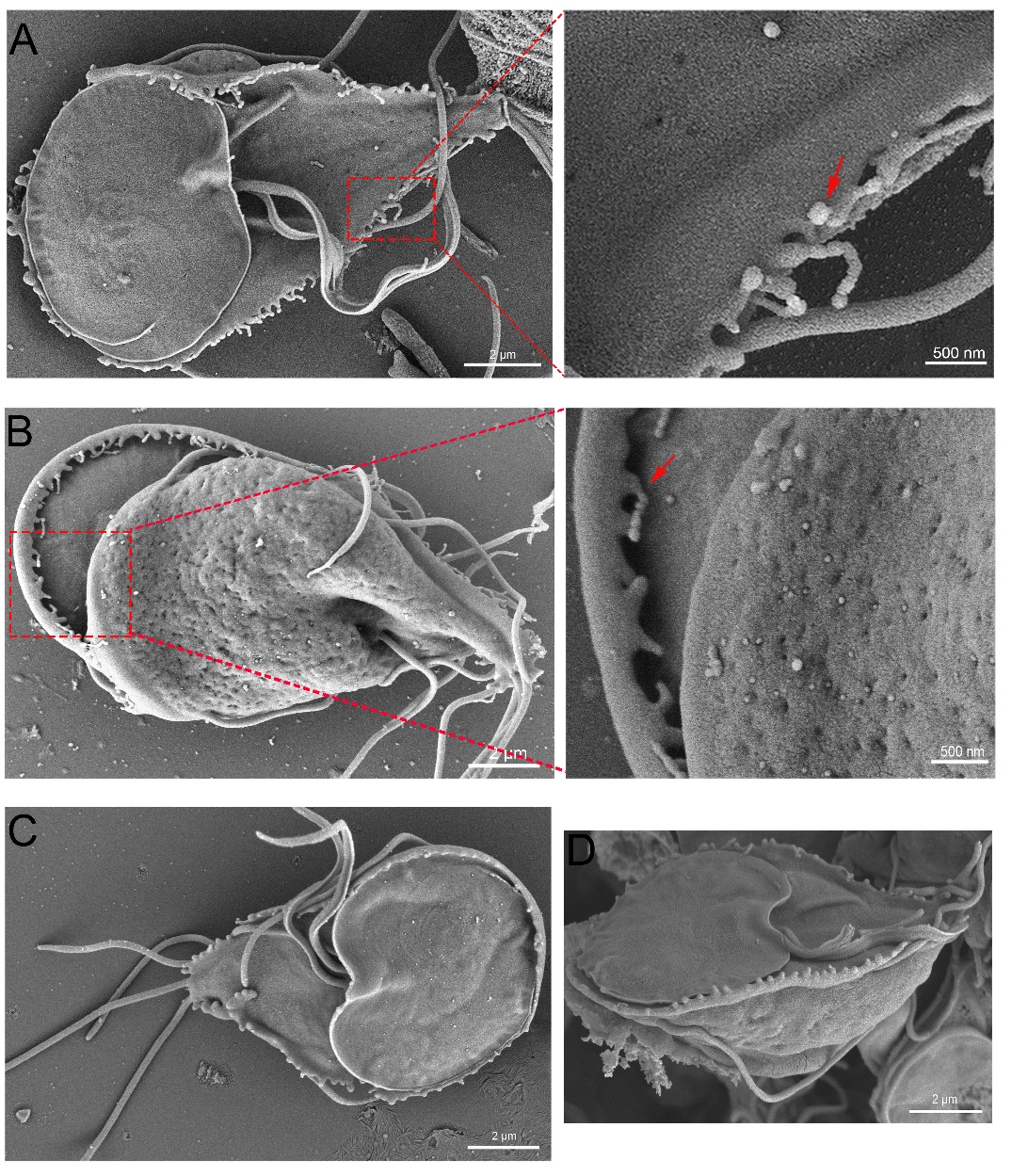


**Supplementary Fig. 2** Observation of extracellular vesicles (EVs) in *Giardia* trophozoites

(A-B) *Giardia*^+GLV^ trophozoites were subjected to SEM after treatment with 1 mM CaCl_2_ for 1 hour, an improved method to enrich EVs derived from *Giardia* [1] .

(C-D) *Giardia* WBC6^-GLV^ trophozoites were observed by SEM.


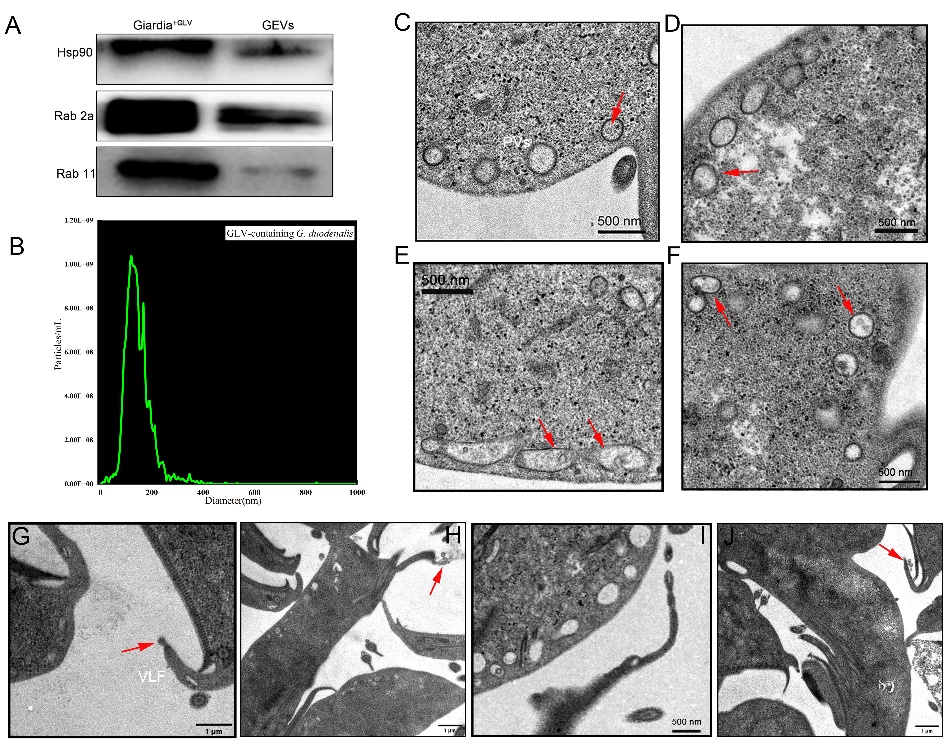


**Supplementary Fig. 3** Observation of extracellular vesicles (EVs) in *Giardia*^+GLV^ trophozoites

(A) Western blot analysis of cell lysates and EVs from the *Giardia*^+GLV^ isolate using polyclonal antibodies against Giardia.

(B) Nanoparticle tracking analysis (NTA) of EVs to show the distribution of particles.

(C-J) The *Giardia* isolate was processed into resin slices and subjected to TEM observation. Transmission electron micrographs showing the presence of EVs in the peripheral vesicles of *Giardia*^+GLV^ parasites (C-F). Visualization of EVs at the end of the ventrolateral flange in the extracellular environment surrounding *Giardia*^+GLV^ trophozoites (G-J).

Red arrows indicate the presence of EVs, and the dashed red squares indicate enlarged regions. Images are representative of at least three independent experiments. The *Giardia*^+GLV^ strain was utilized for these observations.

**The sequencing of GLV whole whole-genome**


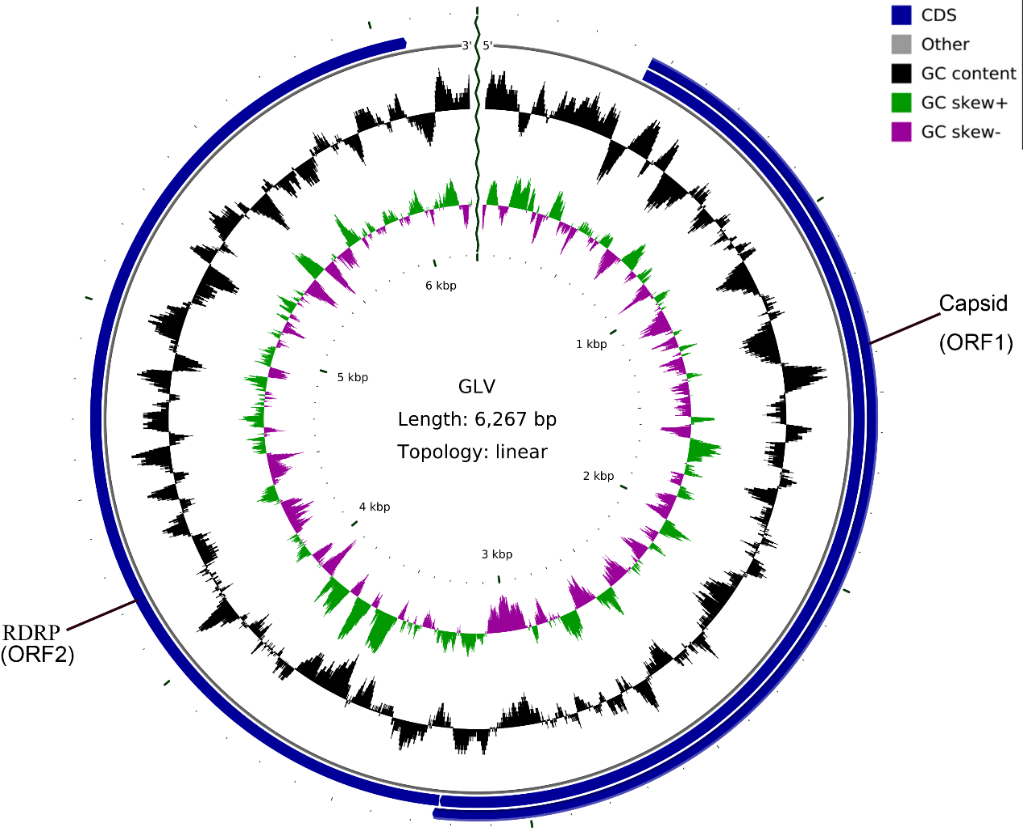


**Supplementary Fig. 4** The whole genome information of GLV virions.

The GLV virions were collected from the supernatant of *Giardia*^+GLV^ culture. The GLV whole-genome was extracted by using a TIANamp virus DNA/RNA kit (Tiangen, Beijing, China). The whole genome shotgun (WGS) strategy was applied to construct libraries with different insertion fragments, utilizing next-generation sequencing technology and the Illumina NovaSeq sequencing platform (250 bp×2) and paired-end reads, Shanghai Personalbio Technology Co., Ltd. (China) carried out whole-genome sequencing. The whole-genome of GLV had been submitted to the NCBI database (GenBank accession number PQ212523), and the capsid (ORF1) primers for GLV were designed based on the whole-genome sequence of GLV. Circle map of the GLV genome. Starting from the inside, the first circle represents the scale, the second circle represents GC Skew, the third circle represents GC content, and the fourth and fifth circles show the positions of CDS, tRNA and rRNA on the genome, respectively. The capsid and the RDRP were showed.

**GLV virions enclosed in EVs**


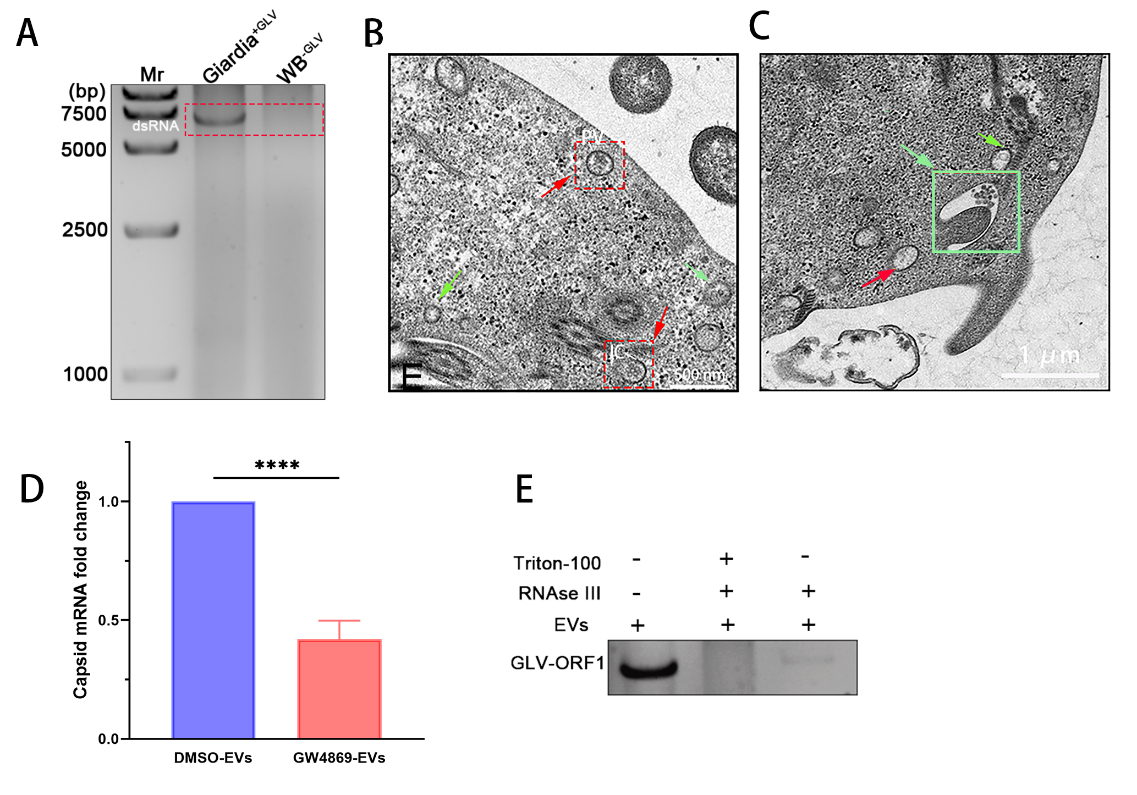


**Supplementary Fig. 5** Characterization of GLV-carried vesicles

(A) The viral genome was extracted using the TIANamp Virus DNA/RNA Kit, verification of *Giardiavirus dsRNA* from WBC6^-GLV^ strain and *Giardia*^+GLV^ strain using agarose gels.

(B-C) Transmission electron micrographs showing GLV virions enclosed within PVs (red arrow), and EVs exsited in PVs (green arrow).

(D) Treatment of the *Giardia*^+GLV^ isolate with the exosome inhibitor GW4869, followed by the collection of EVs to detect the GLV capsid protein. DMSO-EVs regard as the control group that was treated with the same dose of DMSO.

(E) *Giardia*^+GLV^ EVs treated with RNAse III in the presence or absence of Triton X-100; detection of GLV ORF1 (capsid) using RT-PCR.

Green arrows indicate PVs-enveloped EVs, red arrows denote PVs-enveloped, PVs: peripheral vesicles.

**Mice infected with Giardia GS or GS-EVs^+GLV^ trophozoites**


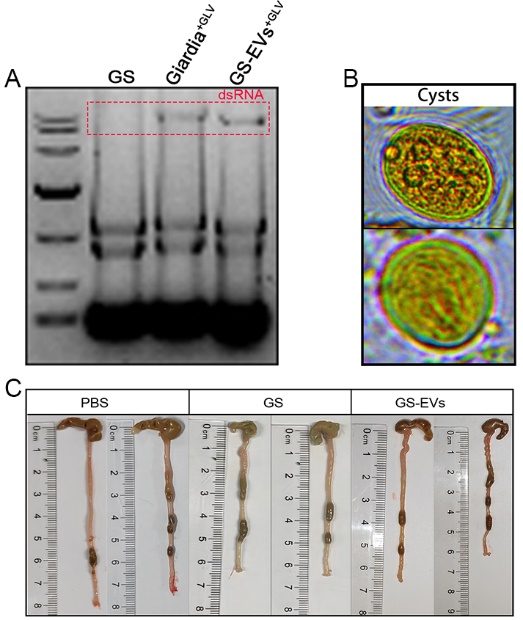


**Supplementary Fig. 6** Impact of GLV-infected *Giardia* on Giardiasis development

(A) *G. duodenalis* GS wild-type trophozoites infected with GLV via EVs, total RNA of trophozoites was extracted and detected by agarose gel. The GLV dsRNA was found in GLV-infected *G. duodenalis* GS trophozoites, is named GS-EVs^+GLV^.

(B) A 100-μl inoculum containing 1.2×10^7^ *G. duodenalis* GS/M trophozoites in PBS that orally administered to 3-week-old C57 female mice, *Giardia* cysts in feces were observed on the fifth day, indicating the successful establishment of the *Giardia*-infected model.

(C) Measurement of the colon of mice at day 14 post-infection, the result show that a shorter colon was observed in *Giardia* GS-infected mice, compared to GS-EVs^+GLV^-infected mice

Table.1

The gene specific primers

| Primers name | Sequence (5′ to 3′) |
| --- | --- |
| TPI- AL3543-F | AAATIATGCCTGCTCGTCG |
| TPI- AL3546-R | CAAACCTTITCCGCAAACC |
| TPI- AL3544-F | CCCTTCATCGGIGGTAACTT |
| TPI- AL3545-R | GTGGCCACCACICCCGTGCC |
| b-giardin-F | AAGCCCGACGACCTCACCCGCAGTGC |
| b-giardin-R | GAGGCCGCCCTGGATCTTCGAGACGAC |
| b-giardin-F | GAACGAACGAGATCGAGGTCCG |
| b-giardin-R | CTCGACGAGCTTCGTGTT |
| GLV-capsid-F | ACTTCCTTTCGAGCTTTAACGTG |
| GLV-capsid-R | TGACAGTTTGGCTCGTGTCA |
| *Giardia* actin-F | CAGAACTGGCGTCAAACGTG |
| *Giardia* actin-R | TTTCCTCCATACCACACGGC |
| FGF15-F | ACATTAAAACAAAAGCGTCAGGA |
| FGF15-R | AAGACATCACCGAAGGCTC |
| IGF-1-F | CCCAATAAATGTTTGTAGCCCTT |
| IGF-1-R | ATTCATTCAAACGCTCTGTCC |

TPI: triosephosphate isomerase; GLV: Giardiavirus; FGF15: Fibroblast growth factor 15; IGF-1: insulin-like growth factor-1.

1. Sana A, Rossi IV, Sabatke B, Bonato LB, Medeiros LCS, Ramirez MI. An Improved Method to Enrich Large Extracellular Vesicles Derived from Giardia intestinalis through Differential Centrifugation. Life (Basel). 2023;13 9; doi: 10.3390/life13091799.
